# Open-source portable solar power supply for plasma generators

**DOI:** 10.1016/j.ohx.2025.e00641

**Published:** 2025-03-17

**Authors:** Md Motakabbir Rahman, Wei Zhang, Ying Zheng, Joshua M. Pearce

**Affiliations:** a Department of Electrical & Computer Engineering, Western University, London, ON, N6A 5B9, Canada; b Department of Chemical and Biochemical Engineering, Western University, London, ON, N6A 5B9, Canada; c Ivey Business School, Western University, London, ON, N6G 0N1, Canada

**Keywords:** Inverter, Open source hardware, Photovoltaic, Solar power supply, Plasma generator, Solar energy

## Abstract

Non thermal plasmas created by dielectric barrier discharge can break down methane directly to constituent elements without carbon dioxide emissions to produce a high-purity hydrogen and byproduct of solid carbon. To fulfill the environmental promise of plasma generators they must be powered with sustainable energy sources such as solar photovoltaic (PV) systems. There is a need to overcome the limitations of past approaches to power plasma systems to develop a completely open source solar PV system design capable of providing the necessary high-quality power. To overcome this research gap, this article provides a customizable open-source PV-powered design for plasma generators, which allows off-grid operation. This design facilitates the modification of existing lab-grade plasma generator setups into portable solar-powered systems. The open source inverter provides for AC loads at both 120 V and 230 V and has an acceptable total harmonic distortion of 3.58 %. The system was successfully able to power plasma generation and produce high quality plasmas with methane. Plasma generators are highly sensitive to any input voltage variation and power oscillation and the open source system provided higher power applied to the plasma reactor, which resulted in increased CH_4_ conversion by 60.5 % and H_2_ production 44.7 % compared to grid supply.

## Graphical abstract

**Figure:**
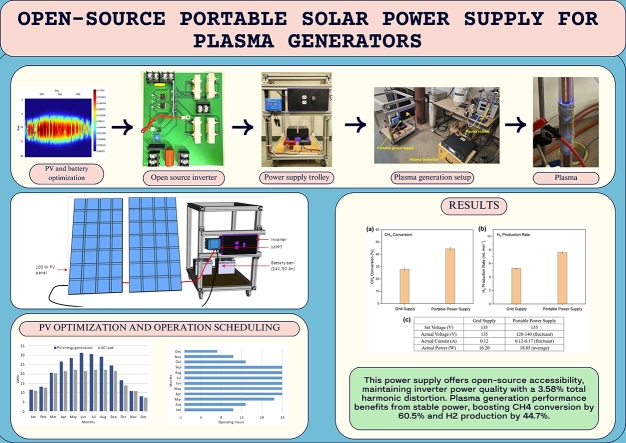

## 1 Hardware in context

Plasma is the fourth state of matter in addition to solids, liquids, and gases. It is a state in which matter is ionized, namely, some of its atoms/molecules have lost or gained electrons. Plasma thus consists of charged particles including positively charged ions and free electrons, leading to a high-reactivity environment. Plasma can also generate high temperatures due to the energy input to the gas for its ionization. The unique properties of plasma make it a valuable tool in various technological applications, depending on the types of plasma.

Plasma can be classified into two basic types. The first type is the thermal plasma, which can be generated at high-pressure (e.g., above 10^5^ Pa) and high-power (e.g., 50 MW) conditions by applying a high-intensity external energy (e.g., electric arc discharge, microwave heating, or laser) to a gas, leading to a high temperature up to 20000 K. Thermal plasma has a wide range of industrial applications:

(1) Welding and cutting [1]. Thermal plasma arcs can provide precise and efficient cutting and welding, particularly for materials that are difficult to cut using traditional methods.

(2) Surface modification of materials [2]. Thermal plasma can be used to modify the surface properties of materials, including plasma-based spraying and coating, where materials can be melted in a plasma jet and then deposited on a substrate for corrosion protection and thermal insulation.

(3) Metallurgical processing [3]. Thermal plasma can be used in metallurgical industries for smelting and refining of metals. The high temperatures generated in plasma allow for efficient processing of various metals.

(4) Waste disposal [4]. Thermal plasma can be used for the destruction of hazardous waste materials. The high temperatures in plasma arc can break down complex organic compounds, converting them into less harmful byproducts.

(5) Treatment of medical waste [5]. Thermal plasma has been developed for medical applications including cutting, combustion, and pyrolysis, utilizing its high temperature under controlled conditions.

(6) Plasma gasification [6]. Thermal plasma can be used for the gasification of organic materials, such as municipal solid waste and biomass. This process can convert waste materials to syngas, which can be further utilized for energy applications.

The second type of plasma is the non-thermal plasma (NTP), also known as cold plasma. It is a type of plasma where the electrons have a much higher temperature than other heavier particles (such as ions and neutral particles). Different from thermal plasma that is featured by its high overall temperatures, there is a significant temperature discrepancy between lighter electrons (thousands of K) and heavier particles (near room temperature) in the NTP. This is because NTP is generated by gas discharge under an applied electric field, where lighter electrons obtain more energy from the external energy input. It should be noted that the role of the NTP is not to provide heat energy to the system, but to generate excited species, allowing starting and enhancing chemical reactions. The lower temperature results in lower energy consumption. Due to these features, NTP has been employed for the following applications:

(1) Surface modification [7]. NTP can clean, activate, and modify materials under mild conditions. This can be used for improving adhesion, wettability, and bonding of coatings and adhesives.

(2) Medical and food industries [8]. NTP has antimicrobial properties, making it effective for sterilizing medical instruments, food surfaces, and packaging materials.

(3) Air and water treatment [9]. NTP can neutralize pollutants and break down organic compounds, thus can be employed for air purification and wastewater treatment.

(4) Synthesis and manufacturing of materials [10]. NTP can be used for the preparation of nano-sized particles, materials, and thin films. The easily controlled conditions of NTP enable precise control of materials properties. It can also be employed in the semiconductor industry for materials processing such as plasma-enhanced chemical vapor deposition (PECVD), allowing for precise deposition of thin films on semiconductor devices like amorphous silicon for solar cells [11].

(5) Generation of high-intensity light [12]. NTP technology-based televisions and lamps use ionized gases to produce high-intensity light. These light sources can be generated efficiently and have the advantage of durability compared to traditional light sources.

(6) Ozone generation [13]. NTP can be used to generate a useful oxidizing agent, ozone (O_3_), for industries because NTP can split oxygen molecules efficiently.

(7) Hydrogen generation. Hydrogen generation can be achieved by various technological routes, including NTP-based water splitting [14], methane splitting [15], ammonia decomposition [16], and reforming of hydrocarbons [17]. NTP can assist these processes via its energetic electrons that collide with molecules, leading to the dissociation of molecules to various fragments and finally forming hydrogen under well-designed routes.

More specifically, technological routes to generate NTP include dielectric barrier discharge (DBD), corona discharges, glow discharge, gliding arc discharge, microwave discharge, capacitively coupled discharge, and inductive coupled discharge [18]. Among them, the DBD uses a dielectric material to create a barrier between electrodes. When an alternating current or a pulsed voltage is applied on the electrodes, the dielectric barrier prevents the formation of continuous discharge. Instead, the discharge occurs in a pulsed and filamentary manner, creating plasma channels during each voltage cycle [19], [20]. Compared with other discharge methods, DBD has advantages of operating at normal atmospheric pressure, low gas temperature, and can be used with many gases.

So far, the combination of DBD and methane splitting for hydrogen production has attracted substantial attention [21], [22], [23], [24]. DBD-induced energetic species can break down methane molecules directly to their constituent elements (carbon and hydrogen). This splitting is also named pyrolysis or cracking. It can be expressed as: CH_4_ → C + 2H_2_. Using DBD plasma for methane splitting offers several environmental benefits. First, it enables reactions at ambient temperature and pressure, unlike traditional high-temperature methods. Second, it reduces greenhouse gas emissions by converting methane into hydrogen and solid carbon without producing CO_2_. Third, it minimizes by-products, requiring less purification and waste management. Lastly, the DBD system is compact, scalable, and can integrate with renewable energy sources like solar cells, making it ideal for sustainable operations. The combination of DBD technology and methane splitting is important due to its ability to generate hydrogen gas at near ambient conditions, avoid greenhouse gases (e.g., CO_2_) emission, and produce a high-purity byproduct of solid carbon. These advantages have encouraged the development of more efficient DBD-based methane splitting technologies in this project.

To fully fulfill their promise plasma generators must be powered with a sustainable source of energy. In the quest for sustainable energy, solar photovoltaic (PV) systems are well established [25], and can provide solutions to power plasma generators of which several pioneering approaches have surfaced. Fang et al. introduced a compact solar-powered plasma generator tailored for DBD applications [26]. The system exhibited versatility by accommodating commercial batteries during insufficient sunlight but faced efficiency hurdles due to a non-optimized PWM-type DC-DC converter and the absence of a zero voltage switching (ZVS) mechanism in the high-frequency switching circuit. Li et al. tackled the challenge with a multi-stage boost converter-driven plasma generator, incorporating a Marx generator for frequency adjustment and demonstrating adaptability to various plasma discharges, but this solution lacked scalability [27]. Ni and colleagues presented an 85 % efficient solar-powered handheld plasma source for microbial decontamination, showcasing impressive microbial reduction, but this approach also lacked scaling abilities [28]. Hafeez et al. proposed a solar-powered decentralized water treatment system, envisioning plasma for industrial wastewater treatment. Despite offering a schematic, the lack of experimental validation leaves questions regarding practicality [29]. These solutions collectively signify strides in solar-powered plasma systems, emphasizing the critical factors of efficiency, scalability, and real-world implementation for advancing this technology. There is a need to overcome the limitations of these approaches to develop a completely open source solar photovoltaic system design [30] that follows best practices for open hardware [31], [32]. To overcome this research gap, this article provides an open-source design and truly customizable solution for plasma generator, which will allow its off-grid application. Furthermore, this design facilitates the modification of existing lab-grade desktop plasma generator experimental setups into portable solar-powered systems.

The key design considerations for a PV system to power the plasma generator was maintaining power quality. This was ensured by monitoring the supply voltage under load conditions and assessing the harmonic distortion.

## 2 Hardware description

**Fig. 1:**
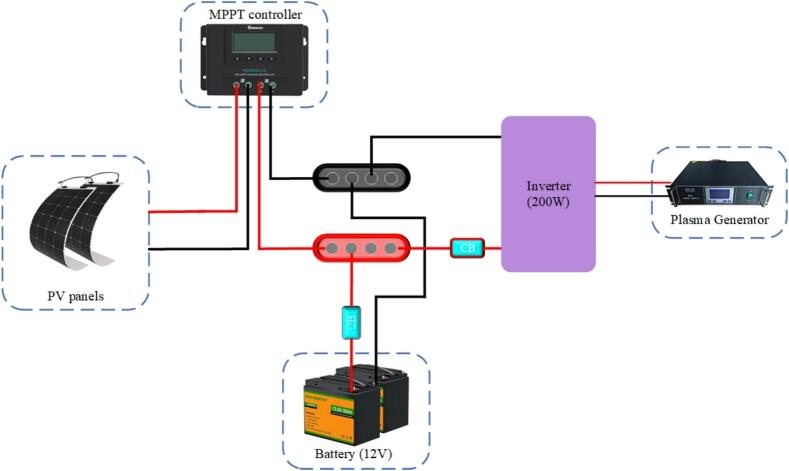
Block diagram of portable solar-powered plasma generation system.

**Fig. 2:**
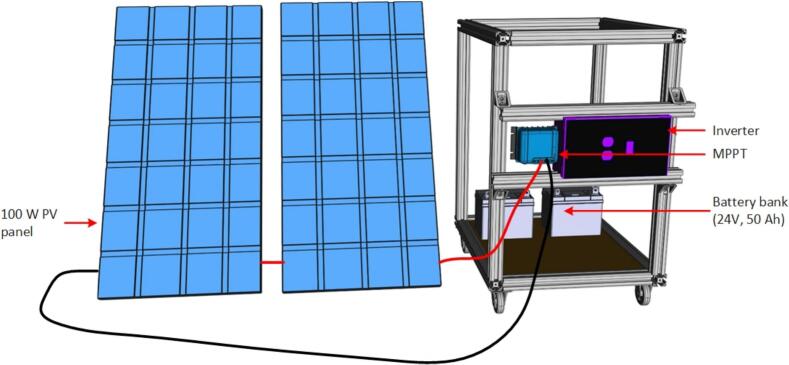
Complete model of portable solar power supply for plasma generator.

First, a setup was devised with the appropriate size of PV modules, batteries, and inverters, along with their protection mechanisms and a wiring diagram as depicted in Fig. 1. Subsequently, to enhance portability and adhere to an open hardware design strategy a specialized inverter was designed for this specific application. Integrated with maximum power point tracking (MPPT) technology and mounted on a single trolley, this inverter enables the development of an application-specific solar-power system tailored for the plasma discharge system (Fig. 2). This system is a compact size, is cost-effective and has an open-source architecture.

An open-source inverter has been designed with a maximum power rating of 500 W, including monitoring the output voltage and observing the total harmonic distortion (THD) present in the output as an assessment of output quality. In this design the inverter will be utilized to directly power the plasma generator, ensuring the quality of the power supply. Observation of the output current and voltages of the plasma generator facilitates the generation of plasma.

A portable power supply solution tailored for operating plasma generators has been developed. The system is characterized by its portability, expandability, customizability, and open-source electronics. The system is intentionally designed as an open-source platform, ensuring adaptability and upgradability. Despite its advanced features, the total development cost is low when compared to proprietary options. The system has the following features:

• A 24-hour power backup capability.
• The systems flexibility is demonstrated through selectable output frequency options (60 Hz/50 Hz) and voltage alternatives (110 V/220 V/230 V).
• Efficient PV energy harvesting is achieved through the utilization of an MPPT charge controller.
• Various protection mechanisms are integrated, guarding against overcurrent, overvoltage, PV short circuits, and battery reverse polarity.
• To prevent overheating, the inverter is equipped with temperature protection and a cooling fan.
• The system's versatility extends to applications beyond plasma generators, accommodating any scenario requiring a portable AC power supply with specific power ratings and off-grid operation.

## 3 Design files

*Low-temperature Plasma Experimental Power Supply (WT2 power supply):*

**Fig. 3:**
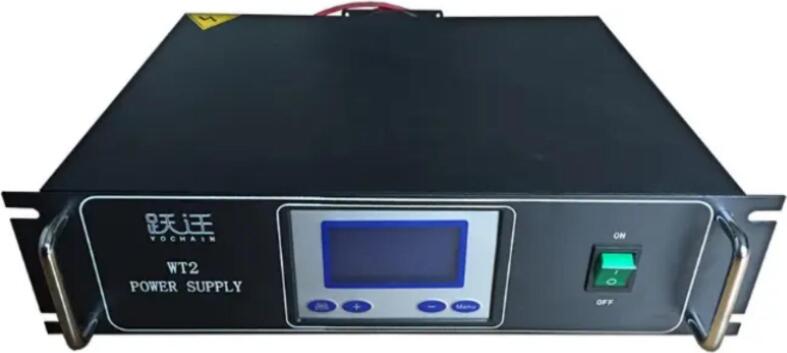
Experimental plasma generator unit (WT2 power supply).

This application involves the use of an experimental plasma generator (WT2 1 kW DC magnetron sputtering plating power supply) (Fig. 3) capable of supporting arc, dielectric barrier, and glow discharge tests under varying atmospheres and pressures. The generator is proficient in creating dielectric barrier discharge and is applicable in diverse gas reactors, including gas–liquid and gas–solid reactors. This plasma experimental featuring a rated input voltage of 230 V (50 Hz) and a capacity of 1kVA. The plasma power supply has an output voltage range of 0–30 kV and a power of 0–500 W. The reactors typically operate within a voltage range of 10–30 kV, a frequency of 8–10 kHz, and a current of 15–45 mA. Optimal conditions for plasma discharge in CO_2_ hydrogenation involve a peak voltage of 15 kV, a frequency of 9 kHz, a current of 22 mA, and a power output of 35 W. The setup also incorporates a dielectric barrier discharge (DBD) reactor.

*PV selection:*

Two Ecoworthy 100 W silicon-based PV modules are used for this project (specifications Table 1). The PV are to be connected in series.

**Table 1:** Specifications of the 100 W PV modules [33].

**Parameter**	**Value (or Range)**
Rated power	100 W
Solar cell	Monocrystalline Si
Maximum peak voltage (V_mp_)	18 V
Open circuit voltage (V_oc_)	21.6 V
Short circuit current (I_sc_)	6.11A
Maximum current (I_mp_)	5.55A
Weight	6.06 kg
Dimension	38.6 × 21.1 × 1.4 in.

*Battery selection and interconnection:*

Two LiFePO_4_ 12 V, 50Ah batteries are used to provide 24hr back up power supply for the plasma generator (specifications in Table 2). LiFePO_4_ batteries are highly suitable for solar applications due to their superior depth of discharge (DOD), extended lifespan, safety and built-in battery management systems (BMS) that ensure safe charging and discharging [34]. Compared to lithium-ion and lead-acid batteries, LiFePO_4_ provides a balanced solution, offering greater safety, stability, and reliability. While lithium-ion batteries offer higher energy density, excelling in longevity and consistent performance, they are ideal for long-term use. Additionally, compared to lead-acid batteries, LiFePO_4_ is more efficient, lightweight, and requires less maintenance, making LiFePO_4_ the preferred choice for off-grid and renewable energy systems. The batteries are to be connected in series and the DC bus voltage in this project is considered 24 V.

**Table 2:** Specifications of the 12 V, 50Ah LiFePO_4_ batteries [35].

**Parameter**	**Value (or Range)**
Rated power	640Wh
Nominal voltage	12.8 V
Voltage range	10–14.6 V
Charge voltage	14.6 V
Maximum continuous charge current	40A
Maximum continuous discharge current	60A
Dimension	8.8 × 5.3 × 7 in.
Weight	4.9 kg

*MPPT selection:*

For tracking the maximum voltage of the PV and charging the 24 V battery setup a 24 V, 20A MPPT was needed. In this regard Victron energy smart solar 100/20, 24 V MPPT is used (Table 3**).**

**Table 3:** Specifications of the 100/20A MPPT [36].

**Parameter**	**Value (or Range)**
Battery voltage	12/24/48 V
Maximum output current	20A
Charging algorithm	4 stage adaptive
Nominal PV power	580 W (24 V)
Maximum PV open circuit voltage	100 V
Peak efficiency	98 %
Dimensions (h × w × d)	100 × 113 × 60 mm
Weight	0.65 kg

*Inverter requirements:*

The WT2 power supply operates on 230 V, 50 Hz AC, while the battery voltage is 24 V. Therefore, an inverter needed to be designed to convert the 24 V DC voltage to AC, and the power quality must be sufficient to operate the plasma generator. The inverter designed for this purpose is based on the EGS002 inverter driver. This inverter, coupled with a transformer, will enable the supply of 230 V AC. Additionally, it features a feedback loop that allows for the control and adjustment of the AC output voltage, ensuring it can be fixed at 230 V. For this application the inverter rating is chosen to be 175 W AC set by the transformer power rating.

*Optimization and operation based on the selected PV panel and battery using SAM 2023.12.17* [37] *:*

**Fig. 4:**
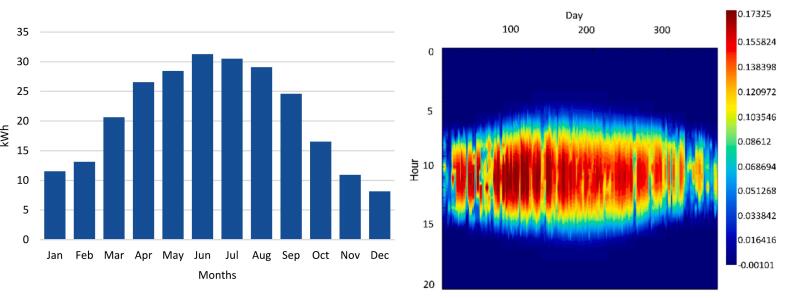
Monthly energy generation of the PV system for year one (left) and annual energy in year as a function of hour in each day (right).

Utilizing weather data specific to London, Ontario, Canada the solar powered system modeling was performed. The chosen PV module, positioned at a fixed tilt angle of 20 degrees, azimuth 180 degrees, and coupled with a 12 V, 50Ah battery, yields the annual PV power generation depicted in Fig. 4.

**Fig. 5:**
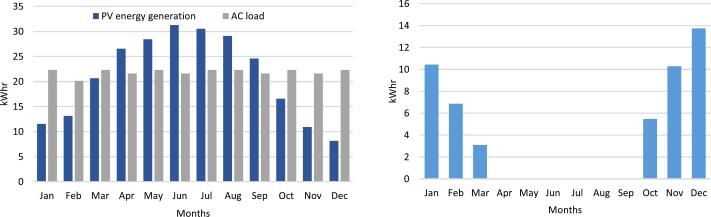
Monthly energy generation versus 24hr running plasma generator power comparison (left) and monthly energy shortage (right).

Upon considering continuous 24-hour operation, as illustrated in Fig. 5, it becomes evident that, from April to September, the portable power supply system sustains uninterrupted plasma generation at a rated 30 W consumption. Conversely, during the remaining months, achieving continuous 24-hour operation for the plasma generator becomes unfeasible, necessitating an optimization of operating hours.

**Fig. 6:**
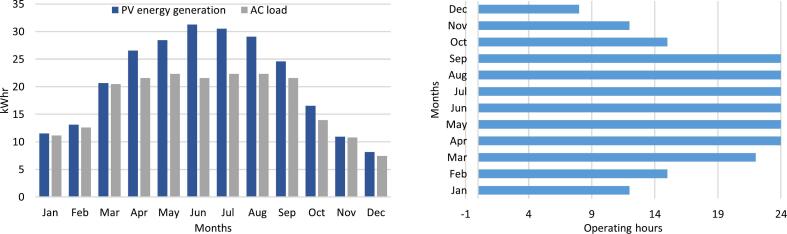
Monthly PV energy generation vs AC load in optimized dispatch (left) and optimized operation of the plasma generator for year one (right).

To attain optimal functionality of the plasma generator under off-grid conditions, the proposed operational schedule (Fig. 6) outlines the most efficient usage pattern. Specifically, during December, the plasma generator operates for the least amount of time (8 h daily), while from September to April, it operates continuously for 24 h.

*Inverter schematic:*

**Fig. 7:**
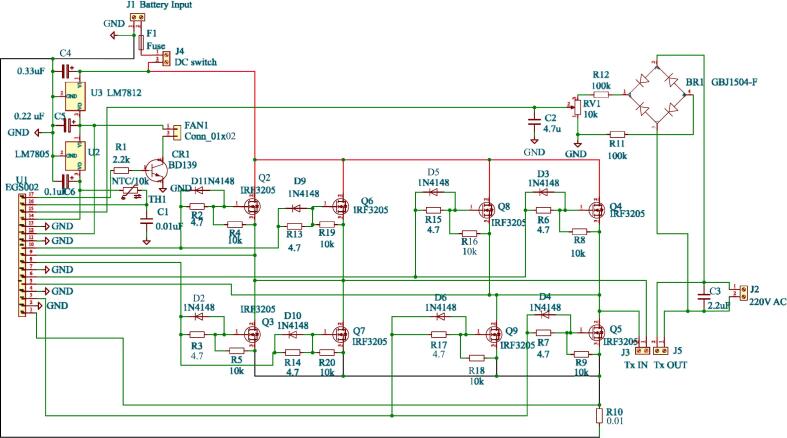
Schematic of 230 V inverter operating on 24 V DC.

The PCB design for the inverter was crafted using KiCAD software (Version 7.0.9) [38]. The schematic (Fig. 7) encompasses the conversion of battery voltage to 5 V and 12 V for inverter controller circuit, a full bridge MOSFET configuration, feedback voltage for the controller, and configuration for temperature control and fan operation.

*5 V and 12 V power supply:*

**Fig. 8:**
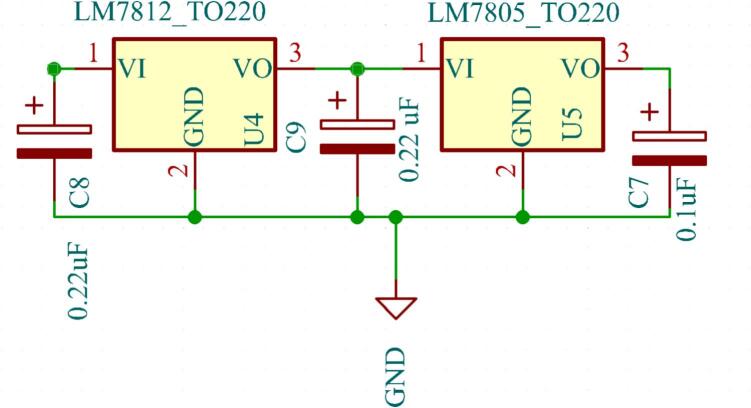
Schematic of 12 V and 5 V supply.

For the inverter driver and the fan, a 12 V and 5 V DC voltage is required at their input. To achieve this, voltage regulators based on two cascaded regulator LM7812 and LM7805 have been employed. The LM7812 regulates the battery voltage to 12 V, and subsequently, the LM7805 regulates the 12 V output from the LM7812 to 5 V. The MOSFET drivers (IR2110) and cooling fan require minimal current (25 mA) directly from 12 V regulator. The EG8010 ASIC draws around 15 mA and auxiliary components from 5 V regulator. Both LM7812 and LM7805 ICs have a maximum current supply capability of 1A, which is sufficient for this application. The schematic is shown in Fig. 8.

*MOSFET selection and interconnection:*

A full bridge structure has been implemented and each MOSFET is expected to carry around 10-25A. To increase the current-carrying limit of the circuit and lower the thermal operating temperature of the MOSFETs, two IRF3205PBF MOSFETs have been included in each switch position of the bridge. This configuration increases the current-carrying limit of the circuit while reducing the risk of temperature rise or high current damage to the MOSFETs.

*AC filter:*

To limit harmonics and ensure the quality of the output AC voltage, a filter has been utilized. It is a first-order filter consisting of one capacitor and inductor (leakage inductance of the transformers). In this application, a 2.2µF ceramic capacitor with a maximum 250 V AC voltage rating has been used, thereby improving the quality of the AC voltage.

*Inverter PCB:*

**Fig. 9:**
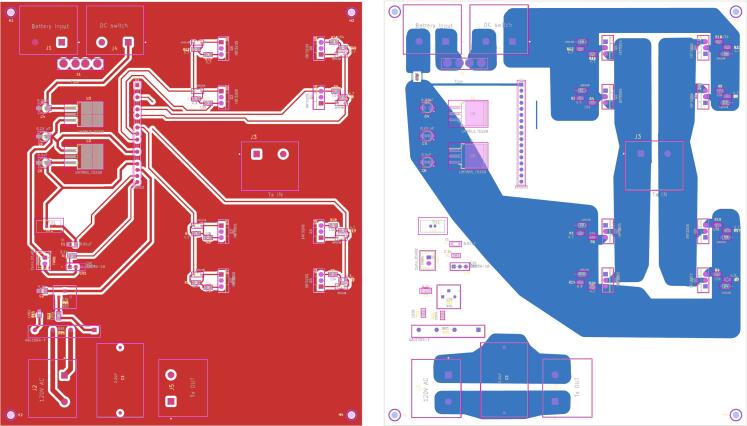
Front and rear PCB layout of the inverter.

**Fig. 10:**
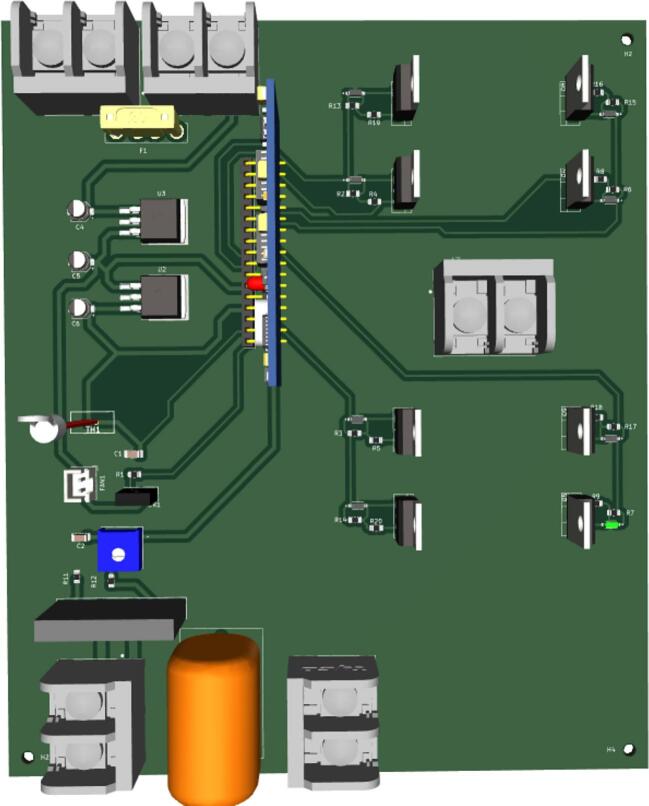
Populated PCB layout of the inverter.

The two-layered PCB of the inverter incorporates both through-hole components and surface-mount components. On the top layer of the PCB, all the controller signals and gate signal traces, while the bottom layer is dedicated to the high current traces of the inverter circuit (Fig. 9). The traces on the bottom layer have intentionally been designed with increased trace width to handle a maximum current of 20A, thus accommodating the inverter's rating of 500 W. The PCB itself measures 178 mm by 152 mm and includes four screw holes for secure attachment to the enclosure (Fig. 10).

*Inverter MOSFET driver:*

**Fig. 11:**
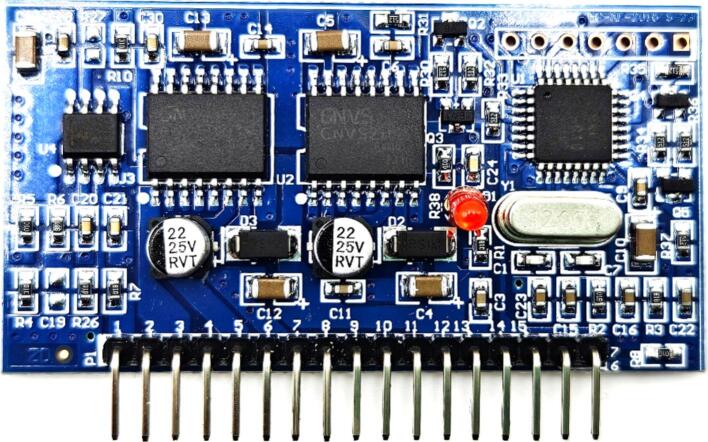
EGS002 Inverter MOSFET driver.

The EGS002 inverter MOSFET driver (Fig. 11) is designed for the development of a single-phase sinusoidal inverter capable of delivering a 230 V/120 V, 50/60 Hz output. It incorporates the EG8010 ASIC as the control chip and the IR2110S high-speed gate driver. The IR2110S efficiently drives both high-side and low-side N-channel MOSFETs, supporting a high-side voltage of up to 500 V. It operates with a gate drive supply voltage range of 10 V to 20 V and is compatible with CMOS and TTL logic levels, with logic input voltage requirements ranging from 3.3 V to 20 V. The IR2110S features fast switching performance includes a typical turn-on delay of 120 ns, turn-off delay of 94 ns, rise time of 25 ns, and fall time of 17 ns, making it suitable for high-frequency applications [39].

The EG8010 control chip supports both unipolar and bipolar sinusoidal pulse-width modulation (SPWM) techniques, with a fixed modulation frequency of 23.4 kHz. Output frequencies are selectable via jumpers, offering standard 50 Hz and 60 Hz options. A notable feature of the EGS002 is its configurable dead time, which is controlled via the EG8010′s hardware control pins (DT1 and DT0). Dead time is essential for preventing cross-conduction in the H-bridge, thereby protecting MOSFETs from damage and reducing switching losses. The dead time can be adjusted to four different settings: 300 ns, 500 ns, 1.0 µs, and 1.5 µs, configurable via jumpers (300 ns: Shorting JP7 and JP8, 500 ns: Shorting JP3 and JP8, 1.0 µs: Shorting JP4 and JP7, 1.5 µs: Shorting JP3 and JP4).

The board integrates comprehensive feedback mechanisms for real-time monitoring and protection. The voltage feedback (VFB) pin interfaces with a resistor-based voltage divider, enabling dynamic adjustments of the output voltage. Current feedback (IFB) provides overcurrent protection by shutting down the output when thresholds are exceeded. Temperature feedback (TFB) controls the fan operation through the FANCTR pin, activating the fan when the temperature exceeds 45 °C and deactivating it when the temperature falls below 40 °C. Default settings for the driver board include short jumpers (JP5, JP2, JP7, JP8) corresponding to 50 Hz output, soft start mode on, and 300nS dead time with a modulation frequency of 23.4KHz.

*Transformer selection:*

**Fig. 12:**
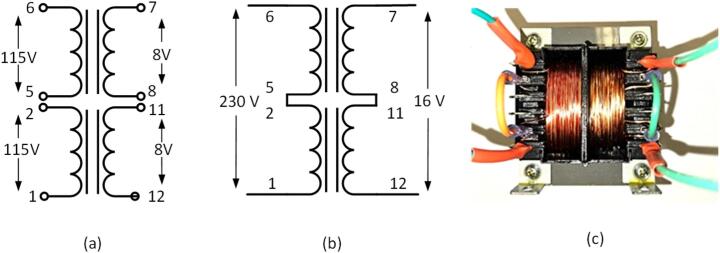
(a)-(c) Transformer connections.

The battery voltage ranges from 24 V to 27 V, permitting an RMS voltage of 17 V at the transformer input. The target output voltage is 230 V. Therefore, the transformer should have a 16 V input and a 230 V output. To meet these requirements, the 185G16 series chassis mount transformer with a capacity of 175VA is employed. It can be wired as illustrated in the Fig. 12, enabling a conversion from 16 V AC to 230 V AC at 50/60 Hz. In making the interconnections, 12AWG wire and wire connectors with heat shrink are utilized.

*3D encloser and parts design:*

**Fig. 13:**
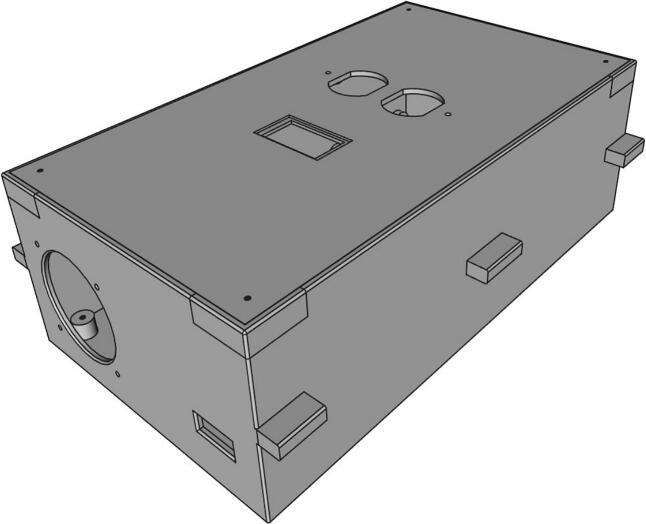
Inverter encloser.

FreeCAD (Version: 0.21.1) [40] was used for designing the enclosure for the inverter Fig. 13. The enclosure dimensions are 300 mm in length, 194 mm in width, and 100 mm in height, facilitating the side-by-side placement of the PCB and the transformer. The enclosure lid is designed with cutouts for the DC switch and dual receptacles for 230 V AC. Additionally, the enclosure features extensions on the sides to seamlessly slide through the side rail of the trolley and securely stay in place on the rails.

**Fig. 14:**
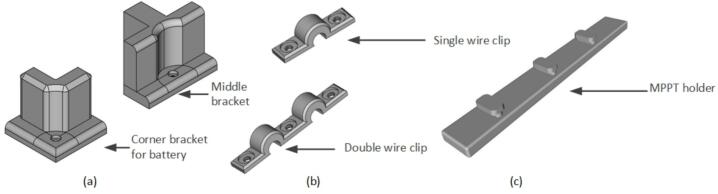
Designs of 3-D printed (a) Battery holder, (b) 10AWG wire clip and (c) MPPT holder.

On the other side, it is essential to 3-D print four side battery holders and two middle holders to secure the batteries in their designated positions. Moreover, several wire clips need to be 3-D printed to ensure the organization and tidiness of the wiring (see Fig. 14).

**Design Files Summary**.

**See**Table 4 summarizes the file type license and location of all the design files.

• Inverter_encloser_lid.stl: The lid of the inverter encloser.
• Inverter_encloser_box.stl: The encloser of the inverter.
• Battery_holder.stl: The battery holder.
• Wire_clip.stl: Double and single wire clip.
• MPPT_holder.stl: MPPT holder.
• FreeCAD 3D parts-FCStd: All 3-D parts in a FreeCAD source file.
• Gerber: A which contains all gerber files necessary for construction of the circuit board.
• Inverter.pro: The main project file for the electrical design
• Inverter.sch: The schematic of the electrical design
• Inverter.kicad_pcb: The board routing for the slice
• Trolley_design.CAD: The mechanical design of the trolley.

**Table 4:** The directory of all the design files.

**Design file name**	**File type**	**Open-source license**	**Location of the file**
Inverter_encloser_lid.stl	STL	CERN OHL-S 2.0	https://osf.io/nksef/files/
Inverter_encloser_box.stl	STL	CERN OHL-S 2.0	https://osf.io/nksef/files/
Battery_holder.stl	STL	CERN OHL-S 2.0	https://osf.io/nksef/files/
Wire_clip.stl	STL	CERN OHL-S 2.0	https://osf.io/nksef/files/
MPPT_holder.stl	STL	CERN OHL-S 2.0	https://osf.io/nksef/files/
FreeCAD 3D parts-FCStd	FCStd	CERN OHL-S 2.0	https://osf.io/nksef/files/
Gerber	Gerber file	CERN OHL-S 2.0	https://osf.io/nksef/files/
Inverter.pro	KiCAD project	CERN OHL-S 2.0	https://osf.io/nksef/files/
Inverter.sch	KiCAD Schematic	CERN OHL-S 2.0	https://osf.io/nksef/files/
Inverter.kicad_pcb	KiCAD PCB	CERN OHL-S 2.0	https://osf.io/nksef/files/
Trolley_design.CAD	CAD	CERN OHL-S 2.0	https://osf.io/nksef/files/

## 4 Bill of materials

*Bill of materials summary:*

The bill of materials (BOM) delineating the specifications for the open-source inverter hardware and PCB is presented in Table 5. The costs associated with the electrical components are notably budget-friendly and can be sourced from a diverse array of vendors. The PCB, meticulously designed using KiCAD, is conveniently reproducible through various suppliers such as PCBWAY and JLCPCB, ensuring cost-effectiveness. Table 5 provides an exhaustive list of readily available components.

**Table 5:** Bill of material for designing the inverter.

**Designator**	**Component**	**Number**	**Price (CAD)/ per unit**	**Total cost (CAD)**	**Source of materials**
J1-J5	Screw Terminal	5	$2.07	$10.35	https://www.digikey.ca/en/products/detail/te-connectivity-amp-connectors/8PCV-02–006/1832526
U1	Inverter Driver Module EGS002	1	$14.40	$14.40	https://www.amazon.ca/Walfront-Inverter-23-4KHZ-Carrier-Frequency/dp/B07TV3BQNJ
C3	Capacitor ECQ-E6225JF	1	$5.77	$5.77	https://www.digikey.ca/en/products/detail/panasonic-electronic-components/ECQ-E6225JF/2567726
Q2-Q9	MOSFET IRF3205PBF	8	$2.69	$21.52	https://www.digikey.ca/en/products/detail/infineon-technologies/IRF3205PBF/812033
CR1	Transistor BD139-10	1	$0.62	$0.62	https://www.digikey.ca/en/products/detail/stmicroelectronics/BD139-10/2827077
TH1	Thermistor 10 k	1	$1.34	$1.34	https://www.digikey.ca/en/products/detail/vishay-beyschlag-draloric-bc-components/NTCALUG01A103JA/12814276
R10	Current Sensing Resistor 0.01 Ohm	1	$0.64	$0.64	https://www.digikey.com/en/products/detail/bourns-inc/CRA2512-FZ-R010ELF/1775029
RV1	10 k Potentiometer	1	$2.47	$2.47	https://www.digikey.ca/en/products/detail/bourns-inc/3386P-1-103LF/1088523
BR1	400 V,15A Rectifier	1	$2.16	$2.16	https://www.digikey.ca/en/products/detail/diodes-incorporated/ GBJ1504-F /815140
D1-D6, D9-D10	Diode 1N4148W	8	$0.13	$1.04	https://www.digikey.ca/en/products/detail/smc-diode-solutions/1N4148W/6022450
C5	0.1 µF, 35 V Capacitor	1	$0.58	$0.58	https://www.digikey.ca/en/products/detail/nichicon/UCB1V0R1MCL1GS/3974069
C4, C6	µF, 35 V Capacitor	2	$0.58	$1.16	https://www.digikey.ca/en/products/detail/nichicon/UCB1VR22MCL1GS/2549603
F1	20A, 32 V Fuse	1	$1.64	$1.64	https://www.digikey.ca/en/products/detail/littelfuse-inc/0ATO020-V/2519106
FAN1	12VDC Fan	1	$8.02	$8.02	https://www.digikey.ca/en/products/detail/sunon-fans/HA30101V3-1000U-A99/10441566
SW	16A, 30 V DC Switch	1	$10.70	$10.70	https://www.digikey.ca/en/products/detail/e-switch/R711FA1-SXXXNBDNNX/4552409
HS	100x 25 x 10 mm Heat Sink	1	$8.99	$8.99	https://www.amazon.com/Antrader-100 mm-Aluminum-Heatsink-MOSFET/dp/B07CVVPC7S
HSP	Heat sink thermal pads	12	$0.19	$2.28	https://www.digikey.ca/en/products/detail/t-global-technology/DC0011-08-TG-A373F-0–25-2A/3466713
U3	12 V Voltage regulator	1	$3.31	$3.31	https://www.digikey.ca/en/products/detail/texas-instruments/LM7812S-NOPB/6110586
U2	5 V Voltage regulator	1	$3.34	$3.34	https://www.digikey.ca/en/products/detail/texas-instruments/LM7805S-NOPB/6110584
Tx	115/230 V, 175VA Transformer	1	$76.84	$76.84	https://www.digikey.ca/en/products/detail/hammond-manufacturing/185G16/454530
AC Socket	AC Power Socket	1	$8.64	$8.64	https://www.amazon.ca/dp/B000U3I1S0
			Total	$185.81	

The 3-D printed parts are crafted utilizing an open-source Modix (BIG-120Z) 3-D printer [41], employing a 1.50 mm hard thermoplastic polymer (polylactic acid (PLA)) filament, with an associated cost of CAD$12.83. It should be noted that any RepRap-class 3-D printer [42], [43], [44] with an appropriate build volume can be used (or the larger components can be printed in sections and assembled using for example the open source Prusaslicer [45]). This design allows for flexibility, as alternative rigid thermoplastic 3-D printing polymers can seamlessly integrate into the project. The CAD files are accessible in STL format as well as the native format from FreeCAD. Additionally, certain components necessary for assembling the portable power supply are produced using the Prusa MK3, another open-source 3D printer, utilizing PLA.

Table 6 lists the components essential for the open-source power supply design. These encompass PV modules, batteries, MPPT, and various protective equipment sourced from diverse suppliers.

**Table 6:** Bill of materials of electrical parts to be purchased for assembly of solar power supply.

**Designator**	**Component**	**Number**	**Price (CAD)/ parts**	**Total cost (CAD)**	**Source of material**	**Material Type**
PV Panels	ECO-WORTHY 100 W Solar Panel	2	$109.99	$219.98	https://www.amazon.ca/ECO-WORTHY-Watts-Volts-Monocrystalline-Solar/dp/B00V4844F4	Semiconductor
Battery	LiFePO4 12 V 50Ah (2 unit)	1	$399.99	$399.99	https://ca.eco-worthy.com/products/lifepo4-12v-50ah-lithium–iron-phosphate-battery	Inorganic
MPPT Solar Charge Controller	MPPT 100 V,20 amp	1	$122.42	$122.42	https://www.amazon.ca/Victron-SmartSolar-MPPT-Controller-Bluetooth/dp/B075NPQHQK	Semiconductor
ANL Fuse	RENOGY 20A ANL Fuse	1	$16.99	$16.99	https://www.amazon.ca/dp/B01LXQWZ7L	Metal
Circuit breaker	T Tocas Waterproof 20A	1	$25.99	$25.99	https://www.amazon.ca/Tocas-Waterproof-20A-Surface-Mount-Breakers/dp/B0BZR8PGGL	Metal
DC bus bar	Jamgoer 300A Bus Bar	1	$42.99	$42.99	https://www.amazon.ca/Jamgoer-Heavy-Duty-Distribution-Terminal-Automotive/dp/B0B55LJDD4	Metal
MC4 connector	Renogy MC4 connector	1	$3.60	$3.60	https://www.amazon.ca/RENOGY-Female-Connectors-Double-Waterproof/dp/B00H1M8ASE	Metal
Wire lugs	Wirefy Tinned Copper Wire	4	$0.25	$1.00	https://www.amazon.ca/dp/B08BYZ2HQK	Metal
Wire	10 AWG	1	$23.99	$23.99	https://www.amazon.ca/TUOFENG-Silicone-Black-Gauge-Stranded/dp/B0CLNV352S	Metal
Screw	M3 self tapping screw	12	$0.042	$0.50	https://www.amazon.ca/OLCANA-Pieces-Tapping-Stainless-Phillips/dp/B0BJKX1X9S	Metal
			Total	$857.45		

To realize the design, the components essential for designing the trolley are detailed in Table 7. The comprehensive cost estimation for the entire project, inclusive of a portable power supply with a one-day power backup, amounts to CAD$1400.

**Table 7:** Bill of material for the trolley.

**Designator**	**Component**	**Number**	**Price (CAD)/ parts**	**Total cost (CAD)**	**Source of material**	**Material Type**
T-Slotted Framing Rail	Four Slot Rail, 40 mm Square, 10ft	3	$81.56	$244.68	https://www.mcmaster.com/6575N25/	Metal
Caster Wheel	Flat-Free 3″ Diameter Rubber Wheel	4	$13.93	$55.72	https://www.mcmaster.com/4941T33/	Metal
Aluminum corner bracket, T nuts and Hex screw	4040 Corner Bracket + M8 T Nuts + M8 Hex Socket	24	$1.87	$44.95	https://www.amazon.ca/Aluminum-Profile-Connector-Bracket-M8x16mm/dp/B0B12G6TFZ	Metal
			Total	$345.35		

## 5 Build Instructions

*Assembly of inverter:*

1. Begin by downloading the entire folder structure from the Open Science Framework (OSF) repository [46] and organize all the components listed in the inverter's bill of materials.
2. For the PCB board, which contains both SMD and through-hole components, it can be ordered from a commercial manufacturer (Table 5), or an open source PCB milling [47], [48], [49] can be used.
3. Start with soldering the SMD components which can be placed and soldered using soldering paste and a heat gun, as illustrated in the Fig. 15. To solder SMD components, gather materials like SMD components, soldering paste, heat gun, circuit board, tweezers, and a hot air rework station. Clean the circuit board, place components, and apply soldering paste. Set the heat gun to 500°F, adjusting airspeed. Hold the heat gun at an angle, melting soldering paste in a circular motion. Observe melting for smooth connections, avoiding direct component heating. Work in intervals, allowing cooling. Inspect joints and touch up if needed. Cool the board naturally to prevent thermal stress.

**Fig. 15:**
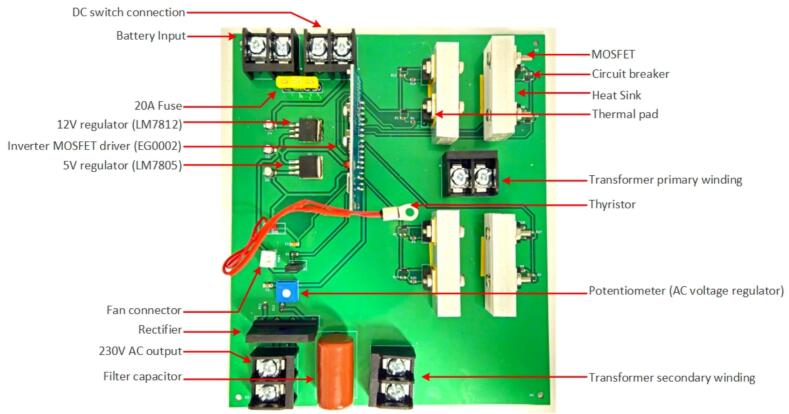
Populated PCB and transformer interconnection.
4. Now place all the through-hole components at the designated holes and solder it from the back using a standard fine-tipped iron.
5. Connect the transformer and the DC switch to the PCB using 12 AWG wire and wire connectors. Ensure proper isolation of connections by using heat shrink.
6. Attach the fan to the PCB using a JST connector.
7. Finally, attach heat sinks to the MOSFET pairs. Cut the heat sink into 50 mm pieces, create corresponding holes, and use heat sink thermal pads and screws to connect the MOSFETs to the heat sink. (Fig. 15)
8. Finally snap in the 20A fuse in the fuse holder.

*Encloser print and assembly:*

1. Use a RepRap-class 3-D printer to 3-D print the enclosure box and its lid listed in the provided Table 4, available on OSF. The enclosure is approximately 300 mm in length, and PLA filament with 15 % infill and 0.20 mm layer height is used for printing.
2. Attach the power receptacle to the lid using the screws and snap the DC switch alongside it.
3. Insert the PCB and the transformer into the base case, following the configuration shown in the Fig. 16 (a). Connect the PCB to the power receptacle and DC switch using 12 AWG wire and wire connectors.

**Fig. 16:**
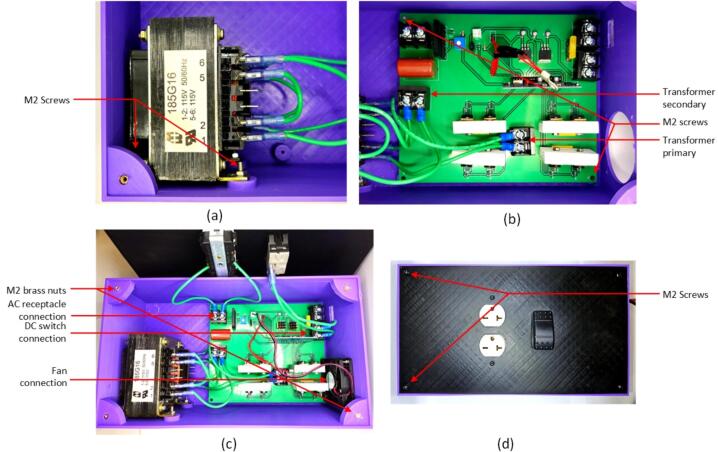
Assembled inverter encloser (a) assembly of transformer, (b) mounting PCB board and connections, (c) top lid connection and (d) encloser connection.
4. Securely place and screw down the fan and the transformer on the side of the enclosure.
5. Finally, install the lid on top of the bottom case part using M2x10 screws.

*Assembly of trolley:*

1. Cut the aluminum frame as per the design (18 in.-2pieces, 27 in.-4 pieces, 24 in.-4 pieces and 21.5 in.- 2 pieces) (Fig. 17 (a)).

**Fig. 17:**
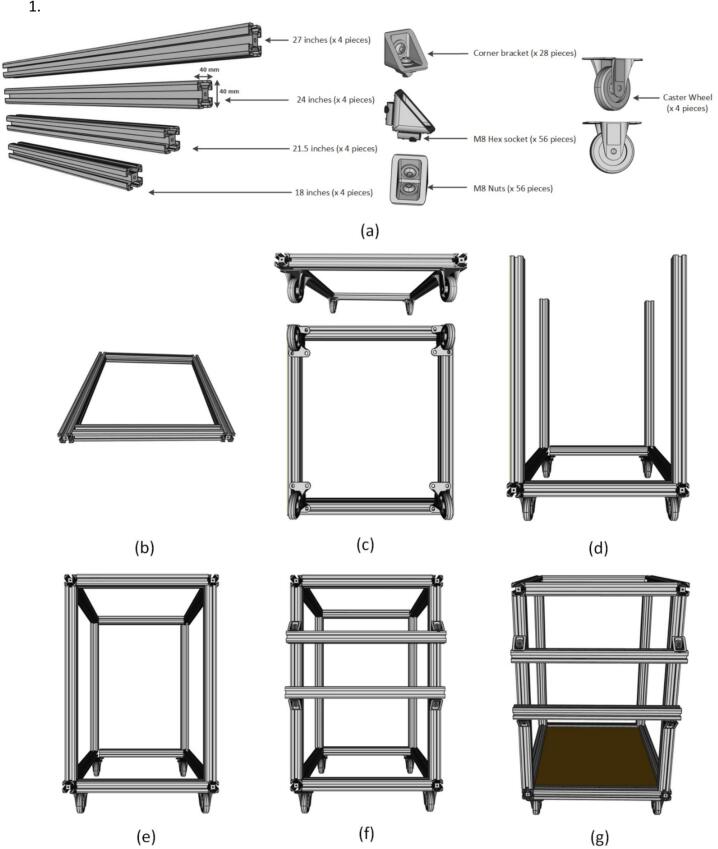
Assembly of trolley (a) Necessary parts, (b) Assemble of the base of the trolley, (c) Assembly of wheels at the base, (d) Assembly of side aluminum frame, (e) Assembly of the top frame, (f) Assembly of side rails and (g) Placement of wooden base.
2. Secure the base frame of 24-inch and 18-inch aluminum extrusion by using corner brackets, screws, and T-nuts (Fig. 17 (b)).
3. Attach the wheel to the base of the trolley (Fig. 17 (c)).
4. Now attach the four side 27-inch aluminum extrusion column using two sets of cornet bracket for each to the base frame (Fig. 17 (d)).
5. Then make the top frame like the base frame and attach it to the side column again using two sets of corner brackets for each (Fig. 17 (e)).
6. Connect two side rails to the trolley for future installation of the inverter and MPPT between the rails (Fig. 17 (f)).
7. Finally, add a wooden base at the bottom to accommodate the battery and protective equipment (Fig. 17 (g)).

*Assembly of power supply unit:*

1. Commence the assembly of the portable power supply unit by downloading and 3D printing the rest of the required parts (battery holder (corner bracket × 4 pieces and middle bracket × 2 pieces), wire clip, and MPPT holder (×2 pieces)) from the OSF using a Prusa MK3 3D printer or similar [50]. Utilize PLA filament with 15 % infill and 0.20 mm layer height during the printing process.
2. Obtain all the components listed in Table 6 bill of materials, which is shown in the Fig. 18.

**Fig. 18:**
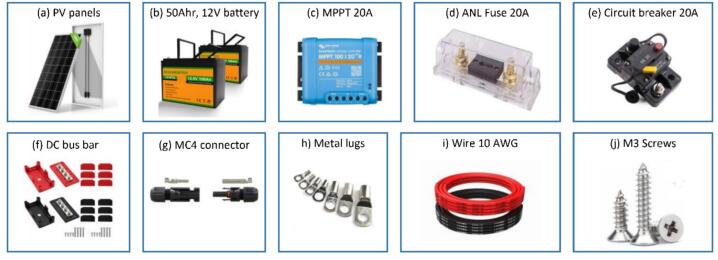
Components to be acquired for assembly of portable power supply: a) PV panel b) 50Ahr, 12 V battery c) MPPT 20A d) ANL fuse 20A, e) Circuit breaker 20A, f) DC bus bar, g) MC4 connector, h) Metal lugs, i) Wire 10 AWG and j) M3 screws.
3. Place the battery on the bottom wooden base and secure it using the battery holder, fastening them to the wood with self-tapping M3 screws (Fig. 19 **a**).

**Fig. 19:**
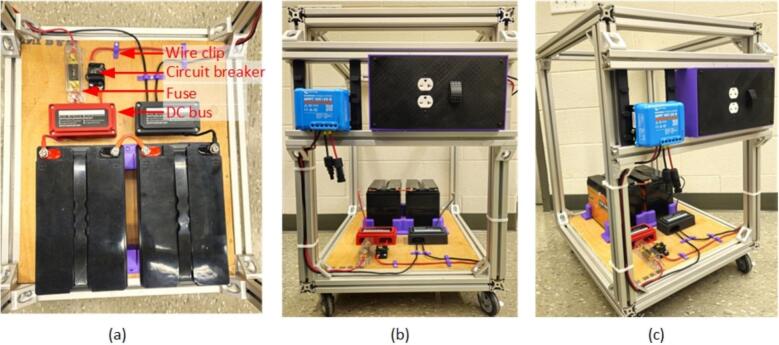
(a) Battery and protection equipment connections (b), (c) Full setup view.
4. Securely position the fuse and circuit breaker on top of the wood and create the necessary interconnections using wire lugs and 10 AWG wires, including the battery interconnection depicted in the Fig. 19 (**a)**.
5. Place the MPPT holder and the inverter on the side rail, sliding the MPPT into its holder (Fig. 20).

**Fig. 20:**
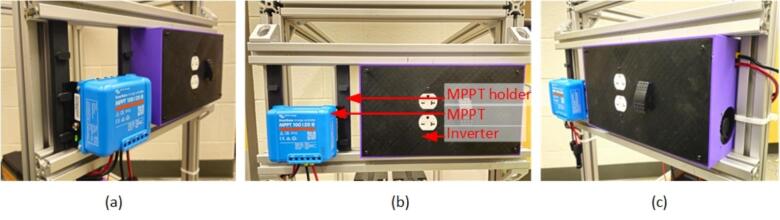
MPPT and Inverter installation (a) left, (b) center and (c) right sides.
6. Establish wire connections between the inverter and the bus bar, as well as between the MPPT and the bus bar, using 10 AWG wire. Use zip ties to secure the wires in place. After establishing the connection with bus bar then connect the MPPT PV ports with MC4 connectors, ready for later PV connections.

*Assembly of DBD plasma system to test open source power supply:*

**Fig. 21:**
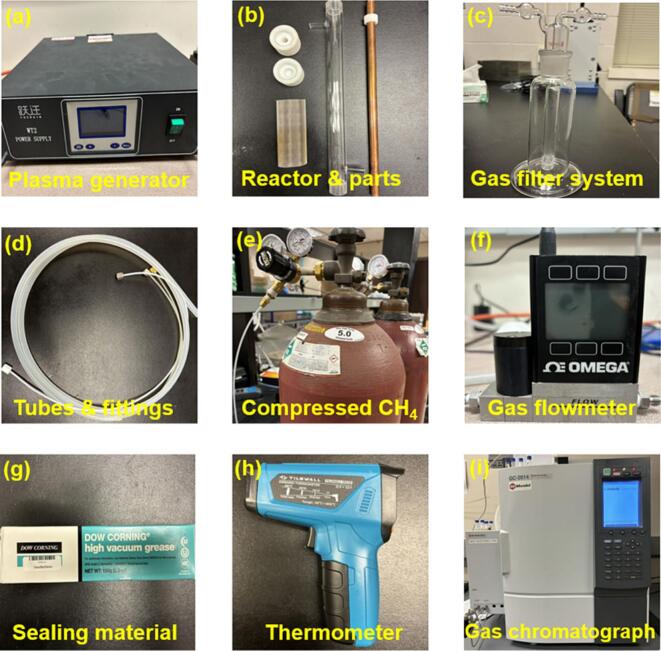
Major components for the assembly of plasma system: (a) plasma generator; (b) reactor tube, electrodes, and related parts; (c) Gas filter system; (d) tubes and fittings; (e) compressed methane gas; (f) gas flowmeter; (g) high vacuum grease for sealing; (h) thermometer; (i) gas chromatograph.

**Fig. 22:**
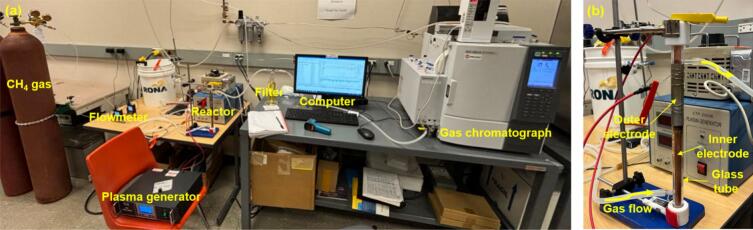
(a) Full view of the assembled plasma test system; (b) Enlarged view of the assembled plasma reactor.

In order to test the open source power supply for plasmas the following experimental assembly was used. Major components for the assembly of the plasma test system are shown in Fig. 21, and a full view of the assembled plasma test system is shown in Fig. 22 (a).

To assemble the system:

Construct the plasma reactor using the parts in Fig. 21 (b). This mainly includes inserting the copper electrode (yellow) into the glass tube, coating the steel-made mesh electrode on the glass tube, and sealing the two sides of the tube using related fittings. There are two small tubes fixed on the reactor acting as the gas inlet and outlet. Specific packing materials and catalysts may be filled into the tube to enhance the plasma generation and promote the methane splitting.
Connect different components using tubes and fittings in the following order: methane gas source, flowmeter, plasma reactor, filter system, and gas chromatograph. All of these components are shown in Fig. 22 (a). The gas chromatograph is connected to the computer system for data acquisition and analysis.
Connect the plasma generator (see Fig. 22 (a)) to the reactor. This includes four steps: (1) Connect the high voltage line of the plasma generator to the inner copper electrode. (2) Connect the ground line of the plasma generator to the steel-made mesh electrode coated on the glass tube (see Fig. 22 (b)). Note that these two lines can be switched because of the alternative pulse property of the plasma generator. (3) There is another line from the plasma generator that must be directly connected to ground for safety. (4) Insert the plug of the plasma generator to a power supply.

## 6 Operation Instructions

*Power supply operation instruction:*

• Begin by connecting the 100 W PV panels in series and then link them to the corresponding PV ports of the MPPT using MC4 connectors.
• Monitor the PV power generation and battery status through the Victron mobile app.
• The setup is now prepared for plasma experiments.
• Initially, connect the power cords of WT2 power supply into the AC receptacles of the inverter and switch on the DC switch.
• Establish all necessary connections between the DBD reactor and gas tanks for the plasma experiments.
• Turn on the WT2 first, then adjust the voltage to generate plasma inside the reactor.

*Plasma system operation instruction:*

**Fig. 23:**
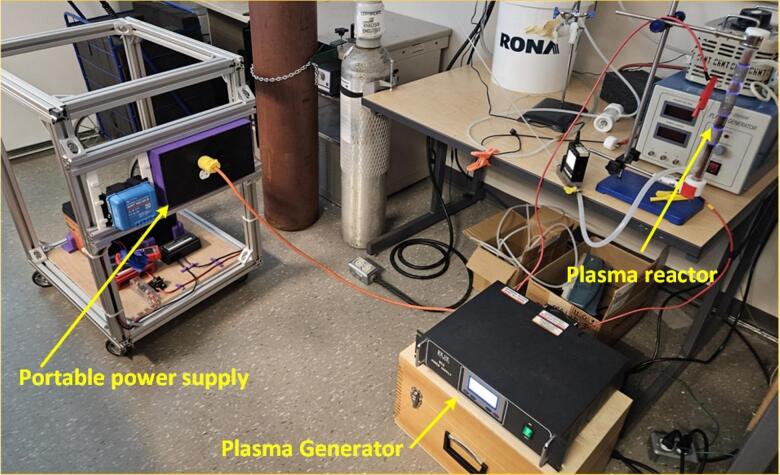
Assembled plasma test system with portable power supply.
• Turn on the gas flowmeter to monitor gas flow, then turn on the methane gas tank. The gas will flow into the plasma reactor, then to the filter, and finally to the gas chromatograph to quantify the produced gas composition.
• Turn on the plasma generator, adjust the applied voltage on the panel to a desired value, and click “run/stop” to start/stop the generation of plasma field in the plasma reactor tube.
• The flowing methane gas will be converted to hydrogen gas and solid carbon under the plasma field. Gas products were collected and analyzed by gas chromatograph. Solid products were deposited on the copper electrode during the reaction, which can be collected after the whole test for further analysis.

• After connecting all the components as shown in Fig. 22, the plasma system is ready to be connected to the PV-powered portable power supply unit through its plug as shown in Fig. 23

*Safety:*

Safety considerations for the portable power supply include electrical risks and those associated with working with plasma.

• All electrical interconnections must be insulated using heat shrink.
• When establishing the final connection between the DC link and MPPT, there might be a spark due to the DC interconnection, so proper electrical gloves and safety glasses are required.
• Before connecting the PV modules to the MPPT, the DC bus/battery must be connected to the MPPT first.
• Although the PCB is made compatible to handle a 500 W AC load, for this application, a 175 W transformer is used, which is sufficient to operate the plasma generator. Under no circumstances, however, should a larger load than this be connected to the system.
• The plasma generator produces high-voltage, high-frequency current to the reactor, which should be handled carefully.

## 7 Validation and Characterization

*Evaluation of the quality of the inverter output voltage:*

**Fig. 24:**
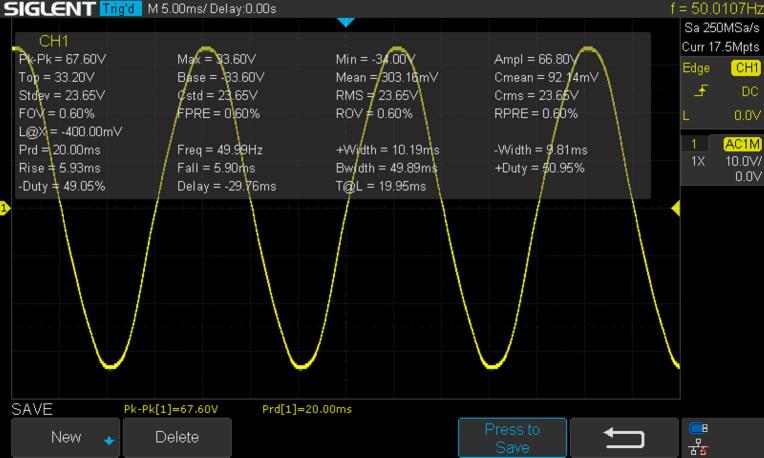
Inverter output voltage (in 10x scale).

The RMS 236.5 V and frequency 50 Hz is seen from the result (Fig. 24) and is summarized in Table 8**.**

**Table 8:** The quality of the output voltage.

**Parameter**	**Value (or Range)**
RMS voltage	236.5 V
Frequency	49.99 Hz
Peak to peak voltage	664 V

*Determination of the total harmonic distortion (THD) of the inverter:*

Total harmonic distortion (THD) is a measure of the harmonic content present in a signal compared to its fundamental frequency. It is commonly expressed as a percentage and is used to quantify the extent to which a signal deviates from a pure sinusoidal waveform. The formula for calculating THD is as follows [51], [52]:

(1) $$THD\left(\%\right)=\frac{\sqrt{{H_{2}}^{2}+{H_{3}}^{2}+\cdots +{H_{N}}^{2}}}{H_{1}}\times 100\left(\%\right)$$

Where H_1_ is the RMS value of the fundamental frequency component and H_2_, H_3_, …, H_N_​ are the RMS values of the harmonic frequency components (second harmonic, third harmonic, and so on).

**Fig. 25:**
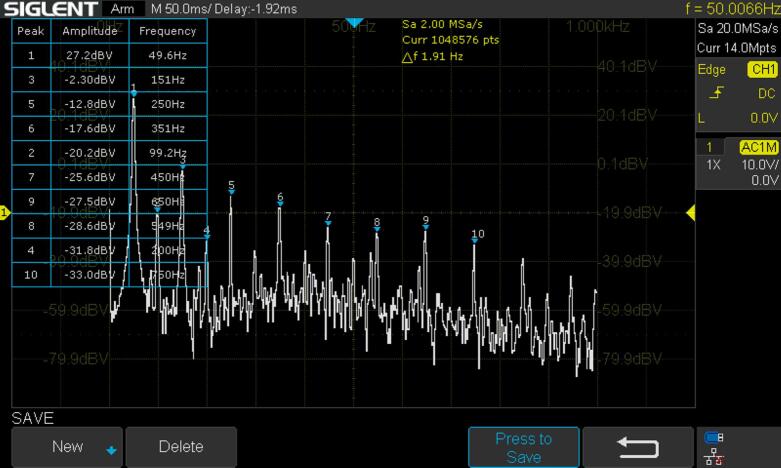
FFT analysis of the output voltage.

Using the total harmonics calculation formula the THD found is 3.58 % (Fig. 25), which is within the limit of the THD requirement for an inverter [53].

*Observation of the glow discharge and color change in plasma reactors:*

**Fig. 26:**
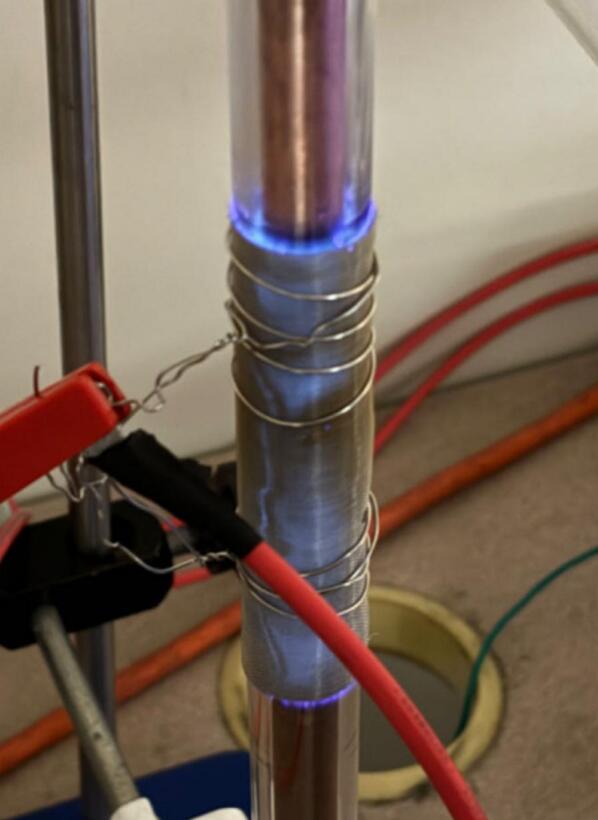
Typical illustration of the purple light generated by plasma-induced gas discharge.

By applying a sufficient voltage on the two electrodes of the plasma reactor, a purple glow can be clearly observed in the plasma generation area (namely, the outer mesh electrode-coated area). The observed light is caused by plasma-induced gas discharge. A typical illustration of the light is shown in Fig. 26.

• *Measurement of the current voltage output of the plasma generator during discharge:*

The voltage value can be set by the panel on the plasma generator. By applying this voltage on the two electrodes of the plasma reactor, the current value can be measured by the plasma generator itself and shown on its screen. Thus, the actual power applied on the plasma reactor can be calculated by the product of the applied voltage and the detected current.

• *Comparative analysis between a grid-connected plasma generator and the portable power supply connected mode.*

The performance of CH_4_ splitting reaction (CH_4_ → C + 2H_2_) in plasma reactors can be evaluated by the following parameters:

(2) $$\;\left(1\right)\;CH4\;conversion\;\left(\%\right)\;=\frac{\mathrm{molesof}{\mathrm{CH}}_{4}input-molesof{\mathrm{CH}}_{4}output}{\mathrm{molesof}{\mathrm{CH}}_{4}input}$$

(3) $$\left(2\right)\;H2\;production\;rate\;\left(mL\;min-1\right)=\frac{\mathrm{molesof}H_{2}produced\left(mol\right)}{gascollectiontime\left(min\right)\times \frac{1}{24500}\left(mol{\mathrm{mL}}^{-1}\right)}$$

Note: the volume of 1 mol gas at 1 atm and room temperature (25 °C) is 24,500 mL.

The moles of input/output gases can be measured using gas chromatograph (Shimadzu Corp., GC-2014). Then, the CH_4_ conversion and H_2_ production rate can be used for evaluating and comparing the performances of the grid-connected plasma generator and the portable power supply-connected plasma generator.

• *A case study of CH_4_ splitting performance measured at various power values:*

**Fig. 27:**
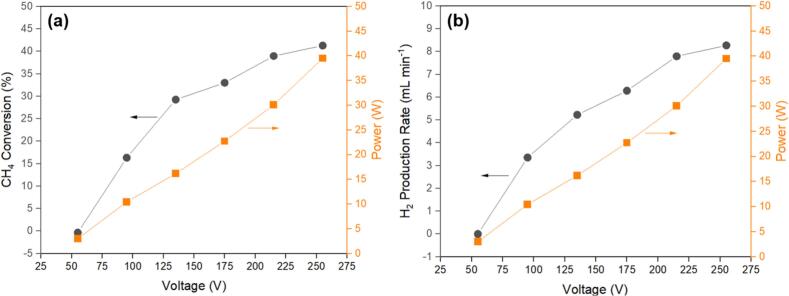
(a) CH_4_ conversion and (b) H_2_ production rate for CH_4_ splitting measured in the DBD plasma reactor at various power values.

The performance of CH_4_ splitting has been measured at various power values using the grid supply. Firstly, a voltage value was set for the plasma generator. By applying this voltage on the DBD plasma reactor, a current value can be obtained. Then, the actual power applied on the plasma reactor can be calculated by the product of the applied voltage and the detected current. Based on this method, a series of voltage values (see X-axis of Fig. 27) were applied on the plasma reactor, and the corresponding power values were calculated and shown on the right Y-axis. Under these conditions, CH_4_ conversion and H_2_ production rate were measured and shown on the left Y-axis. One can see that both CH_4_ conversion and H_2_ production rate increased with the increased power.

• *A comparison of grid supply-generated and portable power supply-generated results:*

**Fig. 28:**
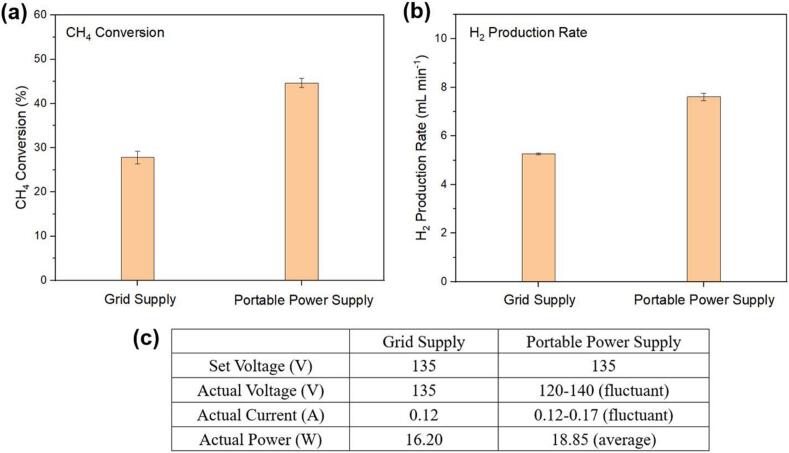
(a) CH_4_ conversion and (b) H_2_ production rate obtained by grid supply and portable power supply. (c) Electrical parameters recorded during the tests.

The performance of CH_4_ splitting was also measured using the portable power supply, and a comparison of the performances generated by the portable power supply and the grid supply is summarized in Fig. 28 (a-b). The initial voltage was set as 135 V for both cases. As seen in Fig. 28 (c), the resultant voltage produced by the portable power supply ranged from 120 V to 140 V, while the generated current ranged from 0.12 A to 0.17 A, resulting in an average power output of 18.85 W. Despite some fluctuations, it should be noted that the current and power values tended to converge towards the stable values obtained from the grid supply. Additionally, the CH_4_ conversion rate reached 44.67 %, with an H_2_ production rate of 7.61 mL min^−1^, both higher values obtained from the grid supply (27.83 % and 5.26 mL min^−1^, respectively).

Two conclusions can be drawn based on the above results. Firstly, the obtained CH_4_ conversion and H_2_ production clearly demonstrated success from using the open source portable PV power supply as the power source for the plasma generator. Secondly, the stability of the portable power supply needs to be further tuned to achieve a stable state and thus deliver a stable voltage value.

## 8 Discussion

This work adds to the body of open source PV development [54], which includes 1) solar-powered devices, 2) devices for monitoring/controlling PV, and 3) tools used to assist PV deployment. Open source solar-powered devices include those meant for off-grid monitoring such as air, noise and light pollution measuring stations [55] or a mobile observatory [56] as well as devices meant to operate off-grid such as a ball mill [57] for scientific work or devices like a drum granulator for seedball production [58]. Open source devices for monitoring and controlling PV include those for IoT PV plant monitoring [59], microgrid automation [60] and performance [61], small PV system monitoring [62], PV device performance [63], and I-V curve tracing [64], [65]. Open hardware has also been developed for a voltage source inverters [66], [67] and batteries [68], [69] (and battery management systems [70]). Finally, other open hardware has been developed to assist in PV deployment such as a climate station for agrivoltaics (that is also solar powered) [71], which can use open source 3-D printed radiation shields [72], sunlight incidence angle measurements [73], [74], as well as a multi-spectral albedometer [75] and hyperspectral measurements [76]. Here this device is both solar powered, but also can play a role in expanding the velocity of PV deployment.

While a dedicated large-scale solution to produce plasma using solar power may not currently exist, there are portable solutions available for harnessing solar energy across various applications. In the realm of energy autonomy, Toshov developed a portable solar power plant [77]. This innovation, featuring a 20-Watt solar panel and a 300-watt inverter, converted sunlight into a symphony of electricity, capable of powering 220 V and 50 Hz devices. The 14Ahr battery, illuminated spaces and powered TVs for up to 4 h, or provided solitary lighting for 20 h. Despite its accomplishments, the absence of maximum power tracking and a clear battery charging method hinted at untapped potential. Chaudhari et. al. introduced an off-grid hybrid online solar power conditioning unit, using a buck converter with MPPT to charge a 48 V battery bank. Equipped with a DSPic controller and IGBT-based inverter boasting over 85 % efficiency, it provided reliable power for those concerned about power uncertainties [78]. Mallwitz of SMA Solar Technology AG showcased the evolution of inverters, from the first generation's 90 % efficiency to SiC-based systems reaching 99 %, dominating the 5 kW power range inverters in the grid-connected market [79]. Minh et. al. designed a 5 kW grid-connected PV smart inverter using a Perturb and Observe (P&O) MPPT algorithm, achieving 89.15 % efficiency and a THD of 4.14 % [80]. Lezcano and fellow architects contributed to an open-source ecosystem with the design and validation of a modular control platform for a Voltage Source Inverter (VSI), capable of supplying 3-phase power to a 10 kW load. Rigorous tests showed a THD level of 5.3 % [66]. The utility of portable solar power supply solutions is evident, considering the shortcomings of existing approaches. While the primary objective is to design a plasma system for hydrogen production, it should be noted that the new design detailed in this study also has broader applicability beyond plasma generators, addressing various use cases requiring 120 V and 230 V AC power. These include powering camp sites, tents, construction sites, mini-clinics, and other experimental installations, promoting flexibility and adaptability in different settings. In addition, the system enables plasma experiments and hydrogen production in off-grid environments, which can be integrated with other hydrogen production sources for energy storage or further applications, such as ammonia production or fertilizer synthesis. Additionally, the plasmas show promise for wastewater treatment, making it highly valuable in remote and resource-constrained areas where clean water access and sustainable energy production are critical needs.

In this portable power supply, the quality of the output voltage is diligently maintained within the recommended 5 % THD limit which ensures that the performance of plasma generators remains unaffected when connected to the portable power supply. While the integration of the plasma generator caused some fluctuations, it led to increased hydrogen production. However, the inverter's bulkiness is a limitation, as its size is dependent on the transformer, with higher power ratings resulting in larger transformers. In contrast, transformer-based inverters offer benefits such as galvanic isolation, enhanced surge protection, and greater robustness. On the other hand, the PV system and battery backup, sustaining a constant 30 W load, operate seamlessly for 24 h between April and September in the case study location in Canada, extending to an average of 12 h for the remaining months. Additionally, comparing the direct grid supply with the portable off-grid supply for plasma generation and hydrogen production reveals higher CH_4_ conversion, resulting in increased hydrogen production, albeit with some fluctuations attributable to the voltage stability issue. Overall, the robust operational profile ensures both portability and reliability of the system. While complete portable off-grid commercial products are currently unavailable, some existing solutions integrate battery and inverter functionalities, though these often come at a premium. Notable examples include the Anker SOLIX C1000 Portable Power Station with a 200 W solar panel, priced at approximately CAD $2,048.00 [81] and the Goal Zero Lithium Yeti 1000X, with a cost of around CAD $1,998.00 [82] excluding any solar panel expenses. It is essential to note that these commercial offerings do not inherently provide a 230 V, 50 Hz supply, a requisite for this specific application. Moreover, the portable power supply inverter possesses the flexibility to be modified for both 120 V/230 V and 50/60 Hz supply, accommodating a wide range of inverter power output ratings, with a maximum capacity of 500 W. Additionally, the PV modules have the capability to be expanded up to 2,000 W, and similarly, the battery bank can be augmented to accommodate larger backup requirements, all seamlessly integrated with the existing trolley infrastructure. This adaptability allows for easy integration into various applications, underscoring the versatility of the open-source system.

## 9 Conclusions

This portable power supply system serves as a versatile solution for various AC loads at both 120 V and 230 V. It is suitable for designing application-specific devices, such as different off-grid portable power supplies for laboratory experiments. The system offers comprehensive guidance and methodologies for designing portable power supplies, encompassing design optimization and hardware implementation. Notably, it provides an open-source solution that is easily accessible and offers a generic solution for diverse applications. The inverter power quality is upheld within an acceptable harmonic range, achieving a total harmonic distortion of 3.58 %. The performance of plasma generation proceeded as anticipated, and the quality of plasma using the portable power supply appears identical. The plasma generator is highly sensitive to any input voltage variation and power oscillation and setting the actual applied voltage to the plasma power supply fluctuated (120–140 V) along with the current (0.12–0.17 A), resulting in higher power (18.9 W) applied to the plasma reactor. This resulted in increased CH_4_ conversion by 60.5 % and H_2_ production 44.7 % compared to grid supply.

## CRediT authorship contribution statement

**Md Motakabbir Rahman:** Writing – review & editing. **Wei Zhang:** Writing – review & editing. **Ying Zheng:** Writing – review & editing. **Joshua M. Pearce:** Writing – review & editing.

## Declaration of competing interest

The authors declare that they have no known competing financial interests or personal relationships that could have appeared to influence the work reported in this paper.
